# Causal Enzymology and Physiological Aspects May Be Accountable to Membrane Integrity in Response to Salt Stress in* Arabidopsis thaliana* Lines

**DOI:** 10.1155/2019/3534943

**Published:** 2019-07-18

**Authors:** Hassiba Bouazzi, Kaouthar Feki, Nabil Zouari, Mouna Sahnoun, Faical Brini, Walid Saibi

**Affiliations:** ^1^Laboratory of Biotechnology and Plant Improvement, Centre of Biotechnology of Sfax/University of Sfax, Tunisia; ^2^Laboratory of Leguminous, Centre of Biotechnology of Borj-Cedria, P. O. Box 901, 2050 Hammam-Lif, Tunisia; ^3^Laboratory of Microbial Biotechnology and Engineering Enzymes (LMBEE), Centre of Biotechnology of Sfax (CBS), University of Sfax, Sidi Mansour Road Km 6, P.O. Box 1177, Sfax 3018, Tunisia

## Abstract

Apart from their significance in the protection against stress conditions, the plant cell membranes are essential for proper development of the diverse surface structures formed on aerial plant organs. In addition, we signal that membrane remodeling and integrity are function of some of causal physiological and enzymological aspects such as the MDA, the ion leakage and also the monitoring of some phytozymes involved in lipid and cellulose metabolisms. Those last ones are related to the membrane structure (lipases and cellulases), that were assessed in durum wheat dehydrin transgenic context (YS, K_1_-K_2_, DH2, and DH4), proline metabolic mutant (P5CS_1-4_) per comparison with the wild-type plant (Wt). We report also the docking data reinforcing the fact that the membrane integrity seems to be function of causal enzymological behaviors, through the molecular dynamic investigation resulting from the dehydrin-phytozyme interactions and also from the inhibition effect of the durum wheat LTP4 on the lipase activity.

## 1. Introduction

At the beginning, it is crucial to note that as much as one-half of the irrigated areas of the world are affected by high salinity. Furthermore, the one distinctive feature of most plants growing in saline environments is that they accumulate increased amounts of low-molecular-weight water-soluble solutes in their cells. The last process is called osmotic adjustment. It has been repeatedly inferred, but never proven, that there might be a relationship between salt tolerance and osmotic adjustment [1–4].

The best characterized biochemical response of plant cells to osmotic stress is the accumulation of organic osmolytes like proline [2, 3, 5, 6]. The accumulation of this atypical amino acid in leaves was first observed [7] and since then its cardinal role as an osmoprotectant effector under various stress conditions, especially under salt stress, has been shown [8]. The effect of salt stress on plants depends on the salt concentration, the duration of exposure, and also the plant genotype [7, 9]. The presence of salt in the environment induces water deficit in plants because of the lowered external water potential, while ion toxicity and nutritional alterations disturb ion transport systems [9, 10]. In addition, salt stress causes membrane damage, alters levels of growth regulators, inhibits some enzymes, and also disrupts photosynthesis, and may thus lead to plant death [2, 11, 12]. It is important to note as well that proline is described as playing catalytic role miming the aldolation reaction [1, 8]. Indeed, the proline-catalyzed aldol reaction mechanism has stimulated considerable debate; at least various mechanisms have been discussed. The Hajos-Parrish-Eder-Sauer-Wiechert reaction represents not only the first asymmetric aldol reaction invented by chemists but also the first highly enantioselective organocatalytic transformation [1, 13].

On the other hand, one of the known plant responses to salt stress is ROS production [5, 12]. Plant cells need to regulate ROS production as excess ROS is potentially harmful to nucleic acids, proteins, and lipids and may therefore lead to cell injury and death [1, 3, 14, 15]. ROS produced through NADPH oxidase activity was shown to be mediated by phospholipid signaling [12, 15]. The second messenger phosphatidic acid is a phospholipid which targets specific proteins to bring about cellular and physiological changes that allow plants to adapt to abiotic stresses [16]. Phosphatidic acid is formed when phospholipase D hydrolyses structural phospholipids at the terminal phosphoesteric bond with release of the hydrophilic head group. In plants, phospholipase D is predominant among the phospholipase families [17]. Their activity upgrades rapidly in response to various environmental stresses like cold, drought, salinity, and wounding ones [11, 18, 19]. It is also very interesting to point out that proline accumulation was shown to be negatively regulated by phospholipase D activity in* Arabidopsis thaliana* [1, 19].

On the other hand, we note that lipid transfer proteins (LTPs) are abundant small members of the family of pathogenesis-related proteins (PR-14) and believed to be involved in plant defense responses. A number of biological roles including antimicrobial defense, signaling, cell wall loosening, and involvement in salt tolerance process acquisition have been proposed and proved. Substantially, LTPs may be important components of direct defense against fungal pathogens and also in some of abiotic stress like salinity [20–22].

Here, the present study deals with the identification of the roles of some physiological parameters and also causal enzymological behaviors that may explain the membrane integrity in case of response of* Arabidopsis thaliana* lines (wild type (Wt), transgenic and metabolic mutant) to salt stress treatment. Hence, we report the potential contribution of phytozymes involved in membrane remodeling and integrity such as lipases and cellulases and also the impact of the tolerance level on the MDA and the ion leakage. Moreover, the* in silico* studies reinforce the role played by transgenic context through the study of the molecular dynamics of the interaction between phytozymes (lipases) and dehydrin or one of these active regions. Eventually, it is basic to note the inhibition role that can be played by the LTP4 during the* in vitro *lipase assessment.

## 2. Experimental Section

### 2.1. Plant Material, Growth Conditions, and Salinity Treatments

*Arabidopsis thaliana* ecotype Columbia (Col-0) is the wild type (Wt) used in this study. Hence, Wt, the transgenic* Arabidopsis* lines overexpressing durum wheat dehydrin* Dhn-5* gene (DH2 and DH4), and the truncated forms (YS, K1-K2) have been previously described by [23–25] and also the proline metabolic mutant lines (P5CS_1-4_) were grown on MS agar medium [26] for one week under light/dark cycle condition of 16-hour light/8-hour dark cycle at 22°C and then transferred to MS medium supplemented or not with NaCl at the concentration of 100 mM. After 8 days of salt stress application, the effect of the addition of NaCl in the medium was determined by measuring some catalytic activities and also some other physiological parameters such as H_2_O_2_ amount, MDA, and ion leakage.

### 2.2. Enzymatic Extract Preparation

Aliquots of frozen fresh shoot material (0.5 g) were ground to a fine powder with liquid nitrogen and homogenized in a cold solution containing 100 mM Tris-HCl buffer (pH 8), 10 mM EDTA (ethylenediaminetetraacetic acid), 50 mM KCl, 20 mM MgCl_2_, 0.5 mM PMSF (Phenyl-Methyl-Sulfonyl-Fluoride), and 2 % (w/v) PVP. The homogenate was centrifuged at 14.000 × g for 30 min at 4°C and the supernatant was used for determination of the enzyme activities [27]. Protein concentration was determined according to Bradford method [28].

### 2.3. Potentiometric pH-stat Assay of Lipase Activities

As indicated by [27], lipase activities were measured potentiometrically at 40°C and pH 8 meaning automatically titrating the free fatty acids released from purified egg L-*α*-phosphatidylcholine (Sigma) as substrate, as previously described [11, 19, 27]. Lipase activities were measured with mechanically stirred triglyceride emulsions [29]. The inhibitory effect of the LTP4 on the lipase activity is monitored by adding the purified durum wheat LTP4 to the reaction mixture at the concentration of 40 *μ*g.mL^−1^.

### 2.4. The Assessment of *β*-Glucosidase Activity

As indicated in materials and methods section of Saibi and Gargouri [30], *β*-glucosidase activity is monitored by incubating 0.2 mL of p-nitrophenyl-*β*-D-glucopyranoside as substrate (in 0.1 M sodium acetate buffer pH 5) with the enzymatic preparation at the appropriate dilution for 15 min at 50°C. The reaction was stopped by adding 0.6 mL of 0.4 M glycine-NaOH buffer pH 10.8. The p-nitrophenol liberated was measured at 400 nm. The molecular extinction coefficient of p-nitrophenol was 18,000 M^−1^.cm^−1^. One unit of enzymatic activity was monitored as the amount of enzyme required to release 1 *μ*mol of p-nitrophenol per min under the assay conditions.

### 2.5. Membrane Lipid Peroxidation Assays

Levels of lipid peroxidation were assessed by measuring the amount of malondialdehyde (MDA) in tissue. Fresh leaf and root samples were homogenized in 10% TCA. The homogenate was centrifuged at 15,000g for 20 min at 4°C. The supernatant was collected and mixed with 0.5% thiobarbituric acid in 20% TCA. Samples were heated at 95°C for 25 min in a water bath and then cooled on ice. The samples were centrifuged at 10,000g for 10 min and the absorbance of solutions at 532 and 600 nm was recorded. The MDA level was calculated using the extinction coefficient for MDA (*ε* = 155 *μ*M cm−1) expressed in nmol MDA g−1 DW [9, 11].

### 2.6. Electrolyte Leakage Assay

Five leaf discs (0.5 cm diameter) were cut from leaves derived from the studied* Arabidopsis thaliana* lines and placed in glass tubes containing 5 ml of deionised water. Thereafter, tubes were kept at room temperature with gentle agitation for 24 h. Electrolyte leakage was determined by measuring the electrical conductivity of the glass tube solution using a conductivity meter and data were expressed as mS.cm^−1^ like described by [31].

### 2.7. Molecular Modeling and Docking Analysis

The unique available sequences of the phospholipid/glycerol acyltransferase (AT4G00400), lipase class 3 family protein (AT1G02660), and thioesterase family protein (AT1G08310) were modeled by the modeling server, phyre 2 (Protein Homology/analogY Recognition Engine V.2). The crystal structure of the 1-acyl-sn-glycerophosphate (lpa)2 acyltransferase, plsc, from* Thermotoga maritima* (PDB: c5kymA) was utilized to model the phospholipid/glycerol acyltransferase. The fungal lipase (PDB: d3tgla) was selected as a template to generate the lipase class 3 family. The crystal structure of murine soluble epoxide hydrolase 2 (PDB: c1cr6A) was used as a structure template for the thioesterase. The dehydrin ORF was modeled using collagen i alpha 1(PDB: c1ygvA) as template. The generated models were then refined by two-step atomic-level energy minimization through the ModRefiner tool available at http://zhanglab.ccmb.med.umich.edu/ModRefiner/[32]. Ramachandran plots were then created by the online tool RAMPAGE (http://mordred.bioc.cam.ac.uk/~rapper/rampage.php) to evaluate and validate models [33]. The PyMol Molecular Graphics System (DeLano Scientific, San Carlos, CA, http://www.pymol.org) was used to visualize the constructed model structure and generate graphical figures. Protein-protein docking of dehydrin and the most three partners' interacting protein was performed using the GRAMM-X server available at (http://vakser.bioinformatics.ku.edu/resources/gramm/grammx) web site [34]. GRAMM-X analyzes the input structures and chooses the best course of action automatically.

### 2.8. Statistical Analysis

Data were analyzed using one-way analysis of variance and treatment mean separations were performed using Duncan's multiple range tests at the 5 % level of significance [35].

## 3. Results and Discussion

At the beginning, it is basic to indicate that membranes are one of the most important structural components of the cell. Hence, they are the protecting layer of the cell bounding the protoplasm and provide the interface for interaction between the outer and the inner components [36]. They have very important functions such as receiving signals, involved in transport of chemicals inwards and outwards. At the same case and in addition to cellulose and hemicellulose, the membrane contains lipids, phospholipids, and galactolipids [11, 27]. In particular, those last compounds were judged playing very important roles on the membrane integrity and also functionality against various biotic and abiotic stresses [11, 31].

On the other hand, it is fundamental to add that durum wheat dehydrin (named DHN-5) is one of the LEA-group 2 family members that was isolated and characterized to be studied and to understand their physiological roles [37]. Furthermore, DHN-5 overexpression in* Arabidopsis thaliana* was realized by our team [23]. Indeed, we proved that DHN-5 confers salinity tolerance to the dehydrin transgenic* Arabidopsis *lines (DHE 2, DH4, and K_1_-K_2_), through the modulation of some of metabolic pathways such as ROS scavenging system, proline metabolism one, and also some of phytozymes like proteases (cysteine and aspartyl proteases) [3, 5].

### 3.1. Rating of the Transcriptional Investigations Leads to Persuasive Salt Tolerance Process

**Figure 1:**
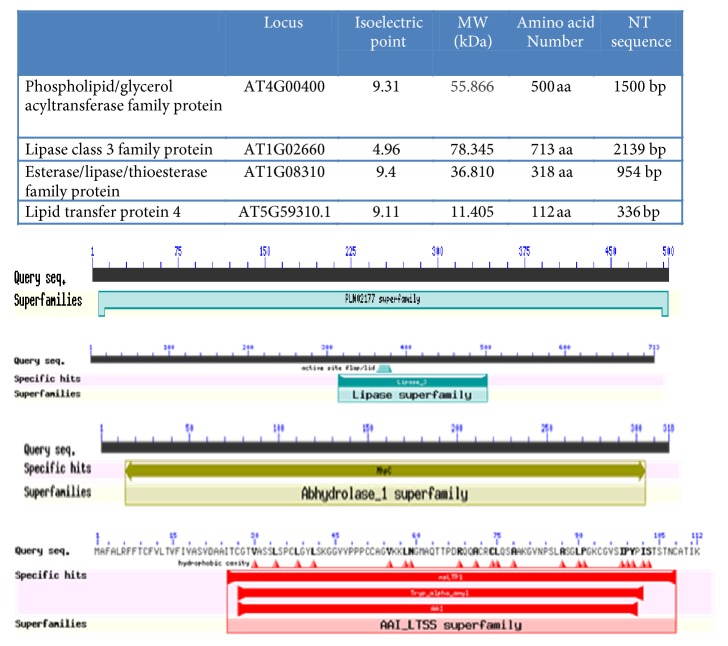
Illustration of the studied phospholipid/glycerol acyltransferase family protein, lipase class 3 family protein, and esterase/lipase/thioesterase family protein properties in the genome of* Arabidopsis thaliana*.

In addition and according to our previous studies at transcriptional level [38], our team proved also the denivelation of 77 genes up or downregulated in DH4 line submitted to salt stress treatment. Indeed, Figure 1 illustrates some properties of phospholipid/glycerol acyltransferase (AT4G00400) which was upregulated as indicated in the list of upregulated genes in transgenic* Arabidopsis *seedlings overexpressing DHN-5 [38] and also lipase class 3 family protein (AT1G02660) and moreover esterase/lipase/thioesterase family protein (AT1G08310) that were downregulated, as indicated in the microarray analysis realized under salt stress and that appeared in the list of downregulated genes in transgenic* Arabidopsis* line (DH4) [38]. Hence, it is important to point out that those three proteins (phospholipid/glycerol acyltransferase, the lipase class 3 family proteins, and the esterase/lipase/thioesterase family protein) are involved in the lipid metabolism that is strongly related to the membrane structure and so with the level of membrane integrity [11]. To achieve this fact, we note that there is another important member named LTP4 (AT5G59310) and it is an upregulated one.

As a point of fact and as indicated in Figure 1, the first one (AT4G00400) is composed of 500 amino acids having an apparent molecular weight of 55.866 kDa and a computed isoelectric point of 9.31. The second one (AT1G02660) consists of 713 amino acids, 78.345 kDa, and a computed isoelectric point of 4.96. The third one (AT1G08310) consists of 318 amino acids, 36.810 kDa, and a computed isoelectric point of 9.4. The last one (AT5G59310) consists of 112 amino acids having a molecular weight of 11.405 kDa and a computed isoelectric point of 9.11.

### 3.2. MDA and Ion Leakage Findings Reflect the Membrane Integrity

**Figure 2:**
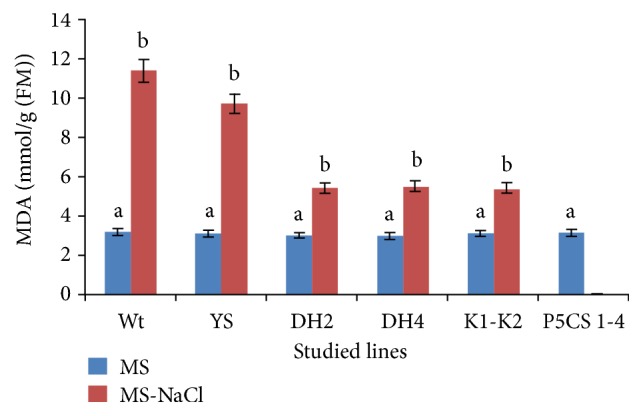
Monitoring of the MDA level of the described* Arabidopsis thaliana *lines (Wt, YS, P5CS_1-4_, DH2, DH4, and K_1_-K_2_) under experimental conditions (conventional medium and salt treated medium). The values represent the means ± SE of three independent experiments. Means denoted by the same letter did not differ significantly at p < 0.05.

It is well established that various abiotic stresses lead to damage to plants through oxidative stress due to the generation of ROS [5, 6]. Thus, the amount of MDA and ion leakage were assessed in both the transgenic* Arabidopsis* seedlings overexpressing DHN-5 full length (DH2 and DH4), YS and K_1_-K_2_ Quiet sequences, Wt and P5CS_1-4_ lines under unstressed and salt stress conditions. In this case and as indicated in Figure 2, MDA content increased obviously in mutant, transgenic, and Wt plants after salt stress. Indeed, the level of MDA in transgenic lines was significantly lower (5.4, 5.5, and 5.4 mmol.g^−1^(FW) in DH2, DH4, and K_1_-K_2_, respectively) than in Wt, YS, and P5CS_1-4_ (11.35 and 9.7 mmol.g^−1^, 0 mmol.g^−1^ (FW), respectively). In addition to this fact, it is crucial to note that the MDA level of the P5CS_1-4_ mutant line under salt stress treatment is considered to be zero because of the inability of the described strain to grow under salt stress conditions used in these studies [3].

**Figure 3:**
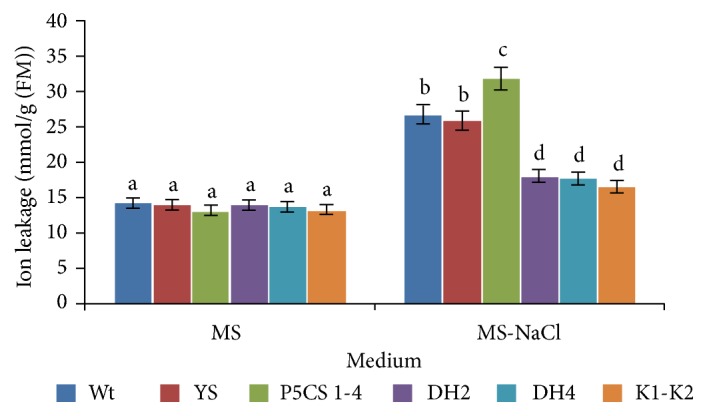
Histogram plot illustrating the electrolyte leakage level of the described* Arabidopsis thaliana* lines (Wt, YS, P5CS_1-4_, DH2, DH4, and K_1_-K_2_) under experimental conditions (conventional medium and salt treated medium). The values represent the means ± SE of three independent experiments. Means denoted by the same letter did not differ significantly at p < 0.05.

On the other hand and as shown in Figure 3, we illustrate the fact that Wt, YS, P5CS_1-4_, DH2, DH4, and K_1_-K_2_ lines presented the same ion leakage level in conventional medium. Under salt stress condition, the Wt, YS, P5CS_1-4_ presented 1.51-, 1.46-, and 1.79-fold higher electrolyte leakage than DH4 line, respectively. Those findings indicated that the transgenic plants produce significantly lower levels of ROS. This fact suggests that overexpression of DHN-5 leads to efficient scavenging system, hence better membrane stability.

### 3.3. Advancement of the Lipase Activity

Table 1 summarizes a clear value reconciliation of the lipase level in full length dehydrin transgenic lines (DH2, DH4), the truncated dehydrin transgenic ones (YS and K_1_-K_2_) compared to the wild-type (Wt) and the proline metabolic mutant (P5CS_1-4_). Indeed, under MS medium Table 1 shows 1.41, 1.40, 1.39, and 1.41 U. mg^−1^ (FM), respectively. At the same case but under salt stress treatment (NaCl 100 mM), the studied activity is more decreased in the case of the transgenic* Arabidopsis* seedlings overexpressing DHN-5 (DH2, DH4, and K_1_-K_2_) which followed 1.30, 1.31, and 1.30 U.mg^−1^ (FM). Concerning both the wild type and P5CS_1-4_, the activity levels were indicated in the table because of the sensitivity of the first one and the hypersensitivity of the second one to grow properly under salt stress. Looking for those findings, it seems that the acquisition of the salt tolerance is accompanied with the decrease of the lipase activity [11]. This fact can be explained through the fact that the membrane structure and integrity must be more protected and this protection seems to be realized via the dehydrin transgenic context by decreasing the lipase activity.

### 3.4. Furtherance of the *β*-Glucosidase Activities

The analysis of data illustrated in Table 2 proves a clear value reconciliation of the *β*-glucosidase level in both used strains such as transgenic lines (DH2, DH4, YS and K_1_-K_2_) in addition to the wild type (Wt) and the proline metabolic mutant (P5CS_1-4_) under conventional standard conditions (MS medium). Indeed, the described lines present 0.52, 0.51, 0.50, 0.49, 0.49, and 0.50 U.mg^−1^ (FM), respectively. At the same case but under salt stress treatment, *β*-glucosidase activity is more decreased in the case of the transgenic* Arabidopsis* seedlings overexpressing DHN-5 (DH2, DH4, and K_1_-K_2_) which followed 0.40, 0.39, and 0.40 U.mg^−1^ (FM). Concerning both wild type and P5CS_1-4_, the activity levels were not really monitored in the table and we stop it to put zero because of the sensitivity of the first one and the hypersensitivity of the last one to grow properly under salt stress. Eventually and according to those results, it seems that it is plausible to explain the fact that the membrane structure and integrity must be more protected and this protection is plausibly realized via the dehydrin transgenic context by decreasing the *β*-glucosidase activity [1].

### 3.5. Interactome Findings Analysis

**Figure 4:**
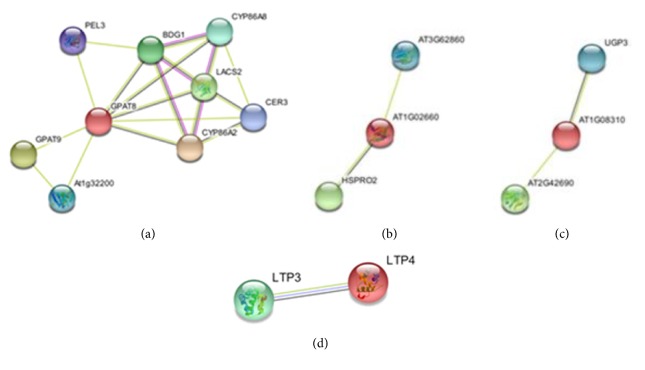
Plots illustrating the interaction of the three studied proteins with the* Arabidopsis thaliana* proteome. (a) The interactome of phospholipid/glycerol acyltransferase family protein, (b) the lipase class 3 family protein one, and (c) the esterase/lipase/thioesterase family. The target proteins are represented with a red sphere.

The study of protein interaction of the three target proteins (phospholipid/glycerol acyltransferase (AT4G00400), lipase class 3 family protein (AT1G02660), and esterase/lipase/thioesterase family protein (AT1G08310)), taken alone, with the proteome of* Arabidopsis thaliana* showed, as follows in Figures 4(a), 4(b), and 4(c), that each one has a range of potential partners, clearly indicated in Tables 3, 4, and 5, respectively. Among phospholipid/glycerol acyltransferase (AT4G00400) partners, we can cite more than a partner involved in metabolism related to tenacity and good membrane structuring and integrity (Table 3). We guess that the positive implication of this enzyme on the tolerance acquisition process in the dehydrin transgenic* Arabidopsis *lines (DH4) is due to their involvement in the maintaining of the membrane stability through the dynamic stability in lipid and phospholipid metabolism in plant [39].

The same case was observed with lipase class 3 family protein (AT1G02660) and esterase/lipase/thioesterase family protein (AT1G08310)) that were downregulated (Tables 4 and 5). Those two enzymes are implicated as downexpressed proteins. Hence, they should have moderate activities in relation to the level of tolerance and sensitivity of the plant under salt stress conditions [39–41]. We can say that the transgenic context gives to the plant a tolerance to salt stress through its multifactorial interactions, following two types of modulation, positive and negative regulations. This modulation may be dictated by the disordered aspect of the DHN-5 and its multifunctionality, already proven [24, 25, 37, 42].

### 3.6. In Silico Studies

To better understand the implication of the transgenic context in the development of the salinity tolerance process through the interaction of DHN-5 with these candidate phytozymes, we used the study of protein-protein interaction. It is within this framework that we have adopted molecular modeling to cool the maximum of data used to reinforce our suggestions.

**Figure 5:**
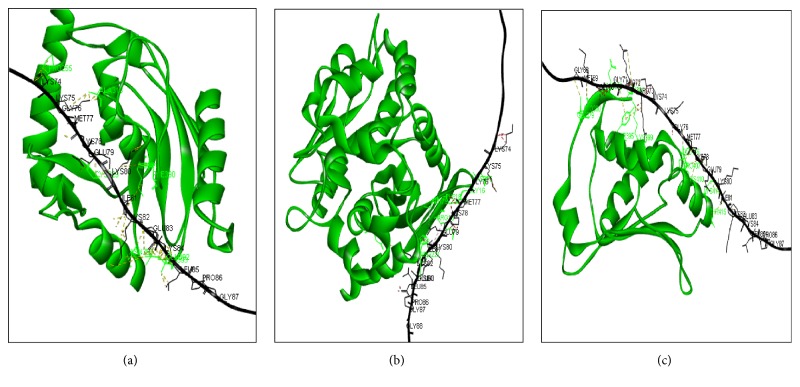
Details of the interaction between phospholipid/glycerol acyltransferase (a), the thioesterase (b), and lipase class 3(c) with dehydrin. The implicated residues were shown as lines. (For understanding of this figure, the reader is referred to Tables 6, 7, and 8, respectively.) The hydrogen bonds were represented by dashed lines.

Accordingly, the phospholipid/glycerol acyltransferase, lipase class 3 family, thioesterase, dehydrin obtained models were refined by ModRefiner and checked by the corresponding Ramachandran plots. Furthermore, the models analysis showed that more than 98% of residues were in the favored and allowed regions. Consequently, these models were validated and retained. Particularly, the dehydrin shows a disordered structure [37]. Moreover, the dehydrin structural model [23, 25, 37] contains the K-segment domain present in two copies as Lys-Ileu-Lys-Glu-Lys-Leu-Pro-Gly sequence. The consensus sequence Asp-Glu-Tyr-Gly-Asn-Pro is also conserved in the N-terminal region as well as the (SSSSSSSS) sequence recognized as the S-(Ser-rich tract) segment. The docking analysis between the dehydrin and those proteins showed that the dehydrin was more effective for lipase class 3 than acyltransferase and thioesterase in terms of interaction hydrogen bonds number (Figures 5(a), 5(b), and 5(c)). Indeed the atomic contact energies of dehydrin-protein complexes were -297 kcal/mol, -148 kcal/mol, while they reached -69.96 kcal/mol for lipase, the acyltransferase, and the thioesterase, respectively. The approximate interface areas of dehydrin for lipase, acyltransferase, and thioesterase were 1316 Å^2^, 1277Å^2^, and 1136 Å^2^, respectively (Tables 6, 7, and 8, respectively). Eventually, those in silico findings reinforce the data collected through the analysis of the transcriptional one realized by our group [38] and moreover argue about the strong implication of causal enzymology in the process of membrane integrity

### 3.7. The Plausible Involvement of LTP4 in Salt Tolerance Process

**Figure 6:**
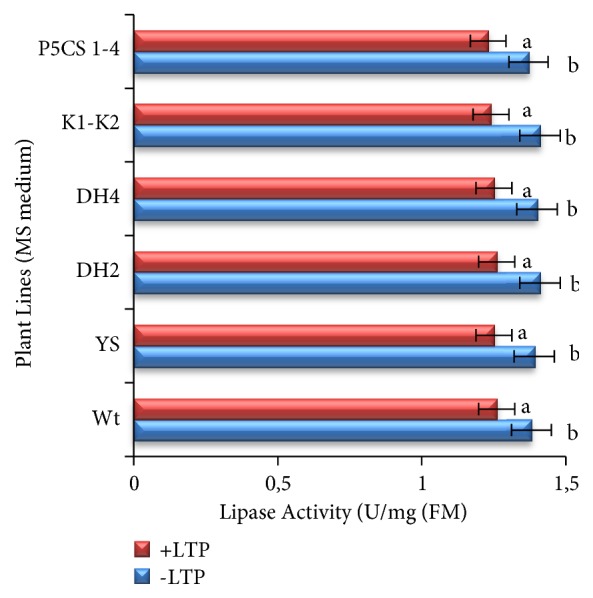
Histogram illustrating the effect of the durum wheat LTP4 on the lipase activity for the leave extracts of described* Arabidopsis thaliana* lines (Wt, YS, P5CS_1-4_, DH2, DH4, and K1-K2) under conventional medium. The values represent the means ± SE of three independent experiments. Means denoted by the same letter did not differ significantly at p < 0.05.

The lipid transfer protein 4 (LTP4) is involved in various biotic and abiotic stresses such as water deprivation, abscisic acid stimulus, and salt stress. It was described also as an inhibitor of some of enzymes involved in lipid metabolism and also as inhibitor of some glycosyl hydrolase ones [20]. In addition, LTP4 represents an upregulated protein as indicated in [38]. Moreover, it is important to signal that the LTP4 was described as an inhibitor of some of enzymes implicated in the carbohydrate and other metabolisms related to the membrane integrity [20]. Based on this fact, it seems that LTP4 can play a protective role during the acquisition of the salt tolerance process by remodeling some of the involved phytozymes in the described process [43]. In this case, it is crucial to test the effect of the durum wheat LTP4 on the lipase activity. Furthermore, the assessment of the last one in presence of the described protein gives birth to the results indicated in Figure 6.

Eventually, the inhibition effect of the durum wheat LTP4 observed on the lipase activity can explain the acquisition of the salt tolerance under salt treated condition (MS-NaCl). Indeed, the decease of the lipase activity under stress condition can protect the membrane structure and so the membrane integrity against the damage that can be caused by the lipolytic activity.

## 4. Concluding Remarks

Eventually and according to the findings followed here, it is fundamental to indicate that the membrane integrity may be explained through some causal enzymological and also physiological aspects due to looking for the acquisition of salt tolerance process in* Arabidopsis thaliana* lines. Given that DHN-5 is a multifunctional protein through its crucial roles played as thermoactivator and thermostabilizator of biocatalysts [5, 37, 42] and also its ability to chelate metals [37], it seems to be a key protein playing a heat protective role and also interacts with multiple partners involved in different pathways (such as proline) and other metabolites (like sucrose and trealose). In addition, through its multifunctionality [37], dehydrin may be a good candidate to interact with the catalysts involved in proline metabolism such as P5CS one. All these findings reinforce the importance of this protein in plant stress response. Finally and to give opportunity to new strategies of research and investigation, we can dare to ask the following question: could proline affect salt tolerance process through a catalytic scene?

## Figures and Tables

**Table 1:** Monitoring of the lipase activity under experimental conditions.

Medium	Plant lines	Lipase Activity (U/mg (FM))
MS	Wt	1.38±0.12 ^a^
	YS	1.39±0.13 ^a^
	DH2	1.41±0.12 ^b^
	DH4	1.40±0.13 ^b^
	K1-K2	1.41±0.2 ^b^
	P5CS_1-4_	1.37±0.18 ^a^

MS-NaCl	Wt	0
	YS	0
	DH2	1.30±0.12 ^c^
	DH4	1.31±0.13 ^c^
	K1-K2	1.30±0.11 ^c^
	P5CS 1-4	0

**Table 2:** Monitoring of the *β*-glucosidase activity under experimental conditions.

Medium	Plant lines	*β*-glucosidase Activity
		(U/mg (FM))
MS	Wt	0.49±0.1 ^a^
	YS	0.50±0.08 ^a^
	DH2	0.52±0.15 ^b^
	DH4	0.51±0.15 ^b^
	K1-K2	0.49±0.1 ^b^
	P5CS_1-4_	0.50±0.1 ^a^

MS-NaCl	Wt	0
	YS	0
	DH2	0.40±0.1 ^c^
	DH4	0.39±0.15 ^c^
	K1-K2	0.40±0.1 ^c^
	P5CS_1-4_	0

**Table 3:** Recapitulation of the first eight partners interacting with the Phospholipid/glycerol acyltransferase family protein in *Arabidopsis thaliana* genome.

Protein	identifier	Annotation
At1g32200	AT1G32200.1	Glycerol-3-phosphate acyltransferase; The enzyme from chilling-resistant plants discriminates against non-fluid palmitic acid and selects oleic acid whereas the enzyme from sensitive plants accepts both fatty acids.

LACS2	AT1G49430.1	Long-chain acyl-CoA synthetase 2; Activation of long-chain fatty acids for both synthesis of cellular lipids and degradation via beta-oxidation. Required for repression of lateral root formation through its role in cutin biosynthesis and subsequent aerial tissues permeability

BDG1	AT1G64670.1	BODYGUARD1

CYP86A8	AT2G45970.1	Cytochrome P450, family 86, subfamily A, polypeptide 8; Catalyzes the omega-hydroxylation of various fatty acids (FA). Acts on saturated and unsaturated fatty acids with chain lengths from C12 to C18.

CYP86A2	AT4G00360.1	Cytochrome P450 86A2; Catalyzes the omega-hydroxylation of various fatty acids (FA). Acts on saturated and unsaturated fatty acids with chain lengths from C12 to C18. Plays a major role in the biosynthesis of extracellular lipids.

GPAT8	AT4G00400.1	Glycerol-3-phosphate acyltransferase; Esterifies acyl-group from acyl-ACP to the sn-1 position of glycerol-3-phosphate, an essential step in glycerolipid biosynthesis

PEL3	AT5G23940.1	PERMEABLE LEAVES3; Required for incorporation of 9(10),16-dihydroxy-hexadecanoic acid into cutin

CER3	AT5G57800.1	ECERIFERUM 3; Involved in cuticle membrane and wax production, and in the typhine and sporopollenin biosynthesis of pollen. Core components of a very-long-chain alkane synthesis complex.

GPAT9	AT5G60620.1	Glycerol-3-phosphate acyltransferase 9

**Table 4:** Recapitulation of the first two partners interacting with the Lipase class 3 family protein in *Arabidopsis thaliana* genome.

Protein	identifier	Annotation
AT1G02660	AT1G02660.1	alpha/beta-Hydrolases superfamily protein
HSPRO2	AT2G40000.1	HS1 PRO-1 2-like protein; Positive regulator of basal resistance
AT3G62860	AT3G62860.1	alpha/beta-Hydrolases superfamily protein

**Table 5:** Recapitulation of the first two partners interacting with the Esterase/lipase/thioesterase family in *Arabidopsis thaliana* genome.

Protein	identifier	Annotation
*AT1G08310*	*AT1G08310.2*	Esterase/lipase/thioesterase-like protein

*AT2G42690*	*AT2G42690.1*	alpha/beta-Hydrolases superfamily protein; catalyzes the hydrolysis of phosphatidylcholine (PC). High activity toward PC, medium activity toward monogalactosyldiacylglycerol (MGDG).

*UGP3*	*AT3G56040.1*	UDP-glucose pyrophosphorylase 3; Involved in the biosynthesis of sulfolipids in the chloroplast. Catalyzes the first committed step in sulfolipid biosynthesis. Converts glucose 1-phosphate to UDP-glucose.

**Table 6:** Comparative analysis of residues involved in the dehydrin interactions with phospholipid/glycerol acyltransferase.

	Name	Distance (Å)	Category	Types	Atomic contact energy (kcal/mol)	Interface area
						(Å^2^)

Acyltransferase	LYS82:NZ -:GLU392:OE1	4,58846	Electrostatic	Attractive Charge	-148	1277
	LYS84:NZ -:GLU392:OE1	5,20883	Electrostatic	Attractive Charge		
	LYS84:NZ -:ASN359:OD1	2,50331	Hydrogen Bond	Conventional Hydrogen Bond		
	LYS84:NZ -:LYS82:O	3,30131	Hydrogen Bond	Conventional Hydrogen Bond		
	LYS82:CE -:GLU392:OE1	3,64848	Hydrogen Bond	Carbon Hydrogen Bond		
	PRO255 -:LYS74	5,01761	Hydrophobic	Alkyl		
	:ALA313 -:LYS75	3,73393	Hydrophobic	Alkyl		
	:CYS329 -:LYS78	3,41551	Hydrophobic	Alkyl		
	:ALA358 -:ILE81	4,2569	Hydrophobic	Alkyl		
	:ILE371 -:ILE81	5,48645	Hydrophobic	Alkyl		
	LYS84 -:LEU393	4,61325	Hydrophobic	Alkyl		
	:PHE390 -:LYS80	5,40312	Hydrophobic	Pi-Alkyl		

**Table 7:** Comparative analysis of residues involved in the dehydrin interactions with the thioesterase.

	Name	Distance (Å)	Category	Types	Atomic contact energy (kcal/mol)	Interface area
						(Å^2^)

Thioesterase	LYS75:NZ -:ASP15:OD2	3,29782	Electrostatic	Attractive Charge	-69.96	1132
	LYS82:NZ -:ASP46:OD1	4,76722	Electrostatic	Attractive Charge		
	:ARG221:NH1 -:ILE81:O	3,37488	Hydrogen Bond	Conventional Hydrogen Bond		
	LYS84:NZ -:SER8:O	3,29526	Hydrogen Bond	Conventional Hydrogen Bond		
	LYS84:NZ -:LYS82:O	3,3014	Hydrogen Bond	Conventional Hydrogen Bond		
	:GLY16:CA -:GLY76:O	2,54704	Hydrogen Bond	Carbon Hydrogen Bond		
	SER79:CB -:GLU79:OE1	3,21836	Hydrogen Bond	Carbon Hydrogen Bond		
	ARG10 -:ILE81	4,6834	Hydrophobic	Alkyl		
	ALA76 -:ILE81	5,04247	Hydrophobic	Alkyl		
	PHE18 -:LYS78	4,68931	Hydrophobic	Pi-Alkyl		

**Table 8:** Comparative analysis of residues involved in the dehydrin interactions with the lipase class 3.

	Name	Distance (Å)	Category	Types	Atomic contact energy (kcal/mol)	Interface area
						(Å^2^)

Lipase class 3	:ARG72:NH1 -:GLU384:OE2	5,27682	Electrostatic	Attractive Charge	-297	1316
	:LYS74:NZ -:GLU384:OE1	3,97953	Electrostatic	Attractive Charge		
	:LYS78:NZ -:GLU446:OE1	4,98943	Electrostatic	Attractive Charge		
	:GLU382:N -:GLY70:O	2,44	Hydrogen Bond	Conventional Hydrogen Bond		
	:LYS410:NZ -:GLY76:O	3,25419	Hydrogen Bond	Conventional Hydrogen Bond		
	:GLY68:N -:GLU379:OE2	3,2076	Hydrogen Bond	Conventional Hydrogen Bond		
	:ARG73:NH1 -:LYS399:O	2,78499	Hydrogen Bond	Conventional Hydrogen Bond		
	:LYS74:NZ -:GLU384:O	3,18541	Hydrogen Bond	Conventional Hydrogen Bond		
	:LYS84:NZ -:LYS82:O	3,30207	Hydrogen Bond	Conventional Hydrogen Bond		
	:LYS410:CE -:MET77:O	3,26563	Hydrogen Bond	Carbon Hydrogen Bond		
	:MET69:CA -:PRO380:O	3,16326	Hydrogen Bond	Carbon Hydrogen Bond		
	:LYS78:CA -:PRO407:O	3,58077	Hydrogen Bond	Carbon Hydrogen Bond		
	:ILE81:CA -:THR415:OG1	3,62323	Hydrogen Bond	Carbon Hydrogen Bond		
	:GLY71:C,O;ARG72:N -:TYR395	4,56177	Hydrophobic	Amide-Pi Stacked		
	:PRO407 -:MET77	3,34072	Hydrophobic	Alkyl		
	:LYS410 -:LYS78	4,48056	Hydrophobic	Alkyl		
	:LYS414 -:ILE81	3,96791	Hydrophobic	Alkyl		
	:TYR395 -:ARG73	4,52373	Hydrophobic	Pi-Alkyl		

## Data Availability

No data were used to support this study.
